# Persistent rise in device-related errors for CGRP monoclonal antibodies: impact of the COVID-19 pandemic and post-pandemic implications

**DOI:** 10.1007/s00210-026-05223-x

**Published:** 2026-03-20

**Authors:** Martina Giacon, Salvatore Terrazzino

**Affiliations:** Department of Pharmaceutical Sciences, University of Piemonte Orientale, “A. Avogadro”, Largo Donegani 2, 28100 Novara, Italy

**Keywords:** Pharmacovigilance, COVID-19, Antibodies, Monoclonal, Calcitonin gene-related peptide, Adverse drug reaction reporting systems

## Abstract

Self-administered calcitonin gene-related peptide (CGRP) monoclonal antibodies (mAbs) are essential therapies, but their safe use depends on adequate patient training. This study examined temporal shifts in the adverse event (AE) reporting profile for CGRP mAbs during and after the COVID-19 pandemic. We conducted a retrospective disproportionality analysis of the FDA Adverse Event Reporting System (FAERS). Reporting odds ratios (RORs) with 95% confidence intervals (CIs) were calculated for the CGRP mAb class across three cohorts: pre-pandemic (2019), pandemic (April 2020–April 2023), and post-pandemic (July 2023–June 2025). Significant changes were defined by non-overlapping 95% CIs. The analysis identified two divergent trends in signals of disproportionate reporting (SDR). There was a significant increase in device-related and use-error AEs, including “drug dose omission by device” (ROR increased from 10.65 to 37.67) and “accidental underdose” (ROR from 0.29 to 16.80). Notably, the SDR for “wrong technique in product usage process” showed continuous intensification across all three periods. Conversely, there was a sustained and significant decrease in reporting of well-established clinical AEs, most notably for “medication overuse headache” (ROR dropped from 56.92 to 2.91) and “constipation” (ROR from 9.86 to 1.61). Reporting patterns shifted dynamically during the pandemic. The surge in use errors suggests vulnerabilities in patient support for self-administered therapies when in-person care is limited, highlighting a critical need for pharmacist intervention and remote education. The concurrent decline in known clinical AEs may indicate shifting reporting priorities. These findings underscore the importance of contextualizing pharmacovigilance data within public health events and warrant further investigation.

**Supplementary Information:**

The online version contains supplementary material available at 10.1007/s00210-026-05223-x.

## Introduction

Migraine is a highly prevalent and disabling neurological disorder, representing a substantial global health burden due to its impact on quality of life, productivity, and healthcare resource utilization (GBD 2021 Nervous System Disorders Collaborators 2024; Dong et al. 2025). The introduction of subcutaneously self-administered calcitonin gene-related peptide (CGRP) monoclonal antibodies (mAbs), including erenumab, fremanezumab, and galcanezumab, marked a paradigm shift in preventive migraine treatment by offering a targeted mechanism with significant efficacy (Cohen et al. 2022; Serra López-Matencio et al. 2022). A key feature of these therapies is their route of administration; they are self-administered biologics delivered via auto-injectors or pre-filled syringes, placing a critical emphasis on patient training and proficiency in device handling for safe and effective use (Stauffer et al. 2018; Mead et al. 2020). Even before the COVID-19 pandemic, challenges in patient training for self-administered biologics were well-documented, with studies highlighting that fragmented or insufficient instruction often led to use errors and reduced treatment adherence (Schiff et al. 2017).

The real-world safety and tolerability profile of the CGRP mAb class has been described as generally favorable (Messina et al. 2023), with common adverse events (AEs) such as injection-site reactions and constipation well-documented since their approval (Holzer and Holzer-Petsche 2022; Sun et al. 2024). Post-marketing pharmacovigilance, which relies on the analysis of large spontaneous reporting systems (SRSs), remains essential for continuously monitoring drug safety, identifying rare or long-term AEs, and detecting changes in reporting patterns (Pacurariu et al. 2015). Disproportionality analysis, using metrics such as the Reporting Odds Ratio (ROR), is a standard and robust methodology within this framework for quantitative signal detection (Rothman et al. 2004; Cutroneo et al. 2024).

The COVID-19 pandemic caused an unprecedented disruption to global healthcare delivery, marked by a rapid and widespread shift to telemedicine (Berry et al. 2022), a reduction in in-person patient consultations (Hatef et al. 2024), and an increase in psychosocial stressors known to trigger or worsen migraine (Hrytsenko et al. 2023). This shift in healthcare practice raised critical questions about the safety monitoring of self-administered therapies, as routine patient education, hands-on device training, and follow-up were significantly affected (Pinzon et al. 2020). As a result, a significant knowledge gap exists regarding how the AE reporting profile for CGRP mAbs may have changed during this period of major healthcare disruption and its aftermath. It remains unknown whether the pandemic generated new safety signals, amplified existing ones related to clinical outcomes or device use, or potentially masked others due to changes in reporting behaviors. Therefore, the primary objective of this study was to characterize and quantitatively compare AE reporting signals for the CGRP monoclonal antibody class across three distinct time periods: pre-pandemic, during the pandemic, and post-pandemic. By analyzing disproportionality in a global pharmacovigilance database, we compared the reporting odds ratios (RORs) and their 95% confidence intervals (CIs) across these periods, defining a statistically significant change in reporting as the absence of overlap between the respective CIs.

## Methods

### Study design and data source

This retrospective pharmacovigilance study used data from the U.S. Food and Drug Administration’s Adverse Event Reporting System (FAERS) database, accessed through the OpenVigil 2.1-MedDRA v24 platform. FAERS is a global spontaneous reporting system that collects post-marketing adverse event (AE) reports from healthcare professionals, consumers, and manufacturers. The reporting of this study follows the recommendations of the READUS-PV statement (REporting of A Disproportionality analysis for drUg Safety signal detection using Individual Case Safety Reports in PharmacoVigilance) (Fusaroli et al. 2024).

We analyzed all AE reports submitted to FAERS for the CGRP monoclonal antibodies erenumab, approved by the FDA on May 17, 2018, fremanezumab, approved by the FDA on September 14, 2018, and galcanezumab, approved by the FDA on September 27, 2018, where at least one of these was designated as the “Primary Suspect” drug. Eptinezumab was excluded from the analysis because its FDA approval in February 2020 precluded a pre-pandemic baseline, and its intravenous administration route represents a significant confounder compared to the self-administered subcutaneous therapies under investigation. To assess the impact of the COVID-19 pandemic on AE reporting patterns, we defined three distinct, non-overlapping temporal cohorts anchored to the official timeline of the global health emergency declared by the World Health Organization (WHO): (a) pre-pandemic cohort: this baseline period included all reports submitted from January 1, 2019, through December 31, 2019, providing a full calendar year of reporting data prior to the global health crisis; (b) during-pandemic cohort: following the WHO’s declaration of a global pandemic on March 11, 2020, this cohort was defined to include reports submitted from April 1, 2020, through April 30, 2023. This end date was precisely chosen to conclude the analysis period immediately before the WHO announced on May 5, 2023, that COVID-19 was no longer a public health emergency of international concern; (c) Post-pandemic cohort: This period began following the official end of the global health emergency, encompassing all reports submitted from the start of the next full quarter, July 1, 2023, through the most recent data available at the time of analysis (June 30, 2025).

For each cohort, AE reports were extracted at the Preferred Term (PT) level according to the Medical Dictionary for Regulatory Activities (MedDRA). To minimize confounding by indication and co-medication, particularly in the during-pandemic cohort, a specific exclusion strategy was implemented. Reports from this period were excluded if they involved co-administration of key drugs strongly associated with the treatment of moderate-to-severe COVID-19. This approach aimed to isolate reporting patterns related to migraine therapy from the overwhelming signal of AEs associated with acute COVID-19 illness and its specific treatments. The excluded medications were remdesivir, tocilizumab, hydroxychloroquine, dexamethasone, and baricitinib. This exclusion was applied only to the during-pandemic cohort, as the context of mass co-administration for COVID-19 was unique to this period and not a significant confounding factor in the post-pandemic era.

### Statistical analysis

A disproportionality analysis was conducted to quantify the reporting frequency of each AE for the CGRP mAb class relative to all other drugs in the FAERS database within each defined period. The primary metric for signal detection was the Reporting Odds Ratio (ROR). This metric assesses the relative proportion of specific adverse events rather than their absolute incidence. Consequently, the ROR is independent of the duration of the observation period or the total volume of reports, as it normalizes the event count by the total number of reports within each respective temporal cohort. The ROR serves as a measure of disproportionality that compares the odds of a given adverse event being reported with a specific drug of interest versus the odds of the same event being reported for all other drugs in the database. An ROR greater than 1 suggests that the adverse event is reported more frequently for the drug under investigation, indicating a potential signal that warrants further evaluation. The ROR, along with its 95% confidence interval (CI), was generated for each drug-event pair using the “Frequentist methods” evaluation tool in OpenVigil 2.1-MedDRA v24 to assess statistical significance. Within this framework, a drug-event pair was classified as a signal of disproportionate reporting (SDR) in accordance with established pharmacovigilance principles (Council for International Organizations of Medical Sciences 2010). The algorithms and criteria utilized to define an SDR are provided in the Online Resource (Supplementary Table 1). An SDR was identified only when all three standard criteria were met simultaneously: (1) a number of reports containing both the target drug and the target adverse event ≥ 3; (2) a lower bound of the 95% CI of ROR greater than 1.0, and (3) a chi-square (*χ*^2^) value ≥ 4 (corresponding to *p* < 0.05), indicating a significant difference between the observed and expected number of reports.

The primary objective was to identify statistically significant changes in reporting signals by tracking how the ROR for a specific adverse event (AE) changed across temporal cohorts. Such variations can indicate the emergence of new safety concerns, reflect the impact of external factors such as the COVID-19 pandemic on reporting behaviors, or show the maturation of a drug’s known safety profile as real-world evidence accumulates. This was assessed by comparing the RORs and their 95% confidence intervals (CIs) across the pre-pandemic, during-pandemic, and post-pandemic periods. A statistically significant change in reporting frequency for a given AE between any two periods was defined by non-overlapping 95% CIs. This comparison was made by determining if the upper bound of the CI for one period was lower than the lower bound for the other period. Additionally, the emergence of new signals was specifically evaluated. An AE was classified as an emerging signal if it was not reported in the pre-pandemic baseline but became a statistically significant SDR in one or both subsequent periods (during and/or post-pandemic). To conduct this comprehensive temporal comparison, the analysis included all Preferred Terms (PTs) reported in association with the study drugs across the three cohorts. The complete results were exported in CSV format for further filtering, comparison, and compilation of the summary tables presented in this study. The overall distribution and overlap of the identified SDRs were visualized using a Venn diagram generated with the online tool Venny, available at https://bioinfogp.cnb.csic.es/tools/venny/index.html. Temporal trends of key adverse event signals were visualized using forest plots generated in Python (version 3.10). For each selected adverse event, the ROR and its corresponding 95% CI for each of the three study periods were plotted. The ROR was displayed on a logarithmic x-axis with a reference line at the null value of 1.0.

## Results

A total of 60,101 potential reports were initially identified in the FAERS database. Following the exclusion of cases involving confounding COVID-19 medications (e.g., remdesivir, tocilizumab) to isolate the migraine-specific profile, a final total of 60,087 reports were included in the analysis. Specifically, the final cohorts consisted of 13,292 reports in the pre-pandemic period, 30,914 in the during-pandemic period (after excluding 14 reports containing COVID-19 specific treatments), and 15,881 in the post-pandemic period. The demographic and clinical characteristics of the cases are summarized in the Online Resource (Supplementary Table 2). Across all three periods, most reports concerned female patients, with the proportion increasing from 66.7% in the pre-pandemic period to 76.7% in the post-pandemic period. The proportion of patients in the 41–64 and ≥ 65 age groups steadily increased over time, while the percentage of cases with missing age data decreased from 62.2 to 30.4%. The most frequently reported outcome in all cohorts was classified as “Other” (non-serious), accounting for over 93% of cases in each period. A notable increase in “life-threatening” outcomes was observed in the post-pandemic cohort (4.2%) compared to the pre-pandemic (0.5%) and during-pandemic (0.3%) periods. Conversely, reports of “hospitalization” decreased from 3.0% pre-pandemic to 0.5% post-pandemic. The USA was the predominant reporting country across all cohorts, though its relative contribution decreased from 95.8% pre-pandemic to 89.8% post-pandemic. Correspondingly, the proportion of reports from other countries, including the United Kingdom and those categorized as “Other,” increased in the post-pandemic period.

The initial analysis identified 199 unique Preferred Terms (PTs) that qualified as signals of disproportionate reporting (SDR) in at least one of the three temporal cohorts. Supplementary Fig. 1 summarizes the distribution and overlap of these SDRs. A core set of 52 SDRs (26.1%) was consistently identified across all periods. The during-pandemic period introduced the largest number of unique SDRs (43, 21.6%), and a substantial overlap of 27 SDRs (13.6%) with the post-pandemic period indicates the persistence of SDRs that emerged during the health crisis. A detailed list of the SDRs for each section of the Venn diagram is provided in the Online Resource (Supplementary Tables 3–9). To further characterize these dynamic shifts, the following sections detail the temporal analysis comparing RORs across periods. This comparative analysis identifies all AEs with a statistically significant change in reporting frequency (non-overlapping 95% CIs), regardless of their SDR status in each period, providing a comprehensive view of the evolving safety profile.

The analysis of adverse event (AE) reporting SDRs across the three periods revealed two distinct and opposing longitudinal trends, as shown in Table 1. There was a consistent and statistically significant increase in reporting for device-related and use-error AEs. Some SDRs, such as “device difficult to use,” increased dramatically during the pandemic (ROR from 0.51 to 48.33) and, despite a partial decline, remained markedly elevated post-pandemic (ROR 36.79). Notably, other critical use-error SDRs not only emerged but intensified over time. For example, the SDR for “accidental underdose” rose from a non-significant pre-pandemic level (ROR 0.29) to a strong SDR during the pandemic (ROR 16.80), and further strengthened post-pandemic (ROR 19.75). A similar pattern of continuous intensification was observed for “wrong technique in product usage process” (ROR increasing from 8.21 to 12.98 across the three periods). Additionally, several SDRs, such as “intercepted product administration error,” emerged as new, strong SDRs during the pandemic and remained high thereafter (ROR 24.38 and 21.03, respectively).

**Table 1:** Temporal evolution of reporting odds ratios (ROR) for adverse events that showed a statistically significant change between at least two of the study periods

Adverse event	ROR pre-pandemic (95% CI)	ROR during-pandemic (95% CI)	ROR post-pandemic (95% CI)
**Device-related and use-error adverse events**			
Accidental underdose	0.29 (0.09–0.89)*	16.80 (15.11–18.67)	19.75 (18.13–21.51)
Circumstance or information capable of leading to medication error	NR	6.49 (5.88–7.18)	8.94 (8.10–9.86)
Device difficult to use	0.51 (0.21–1.24)*	48.33 (46.68–50.04)	36.79 (35.41–38.23)
Device leakage	0.99 (0.61–1.59)*	4.64 (4.16–5.18)	4.80 (4.08–5.64)
Device use error	1.16 (0.76–1.77)*	6.87 (6.14–7.68)	3.56 (2.89–4.39)
Drug dose omission by device	10.65 (8.61–13.17)	37.67 (35.94–39.48)	25.21 (24.26–26.19)
Incorrect dose administered by product	2.95 (1.21–7.20)	15.40 (11.53–20.58)	11.56 (7.57–17.66)
Incorrect product formulation administered	2.43 (0.60–9.92)*	40.41 (28.98–56.34)	74.60 (50.17–110.95)
Intercepted product administration error	NR	24.38 (21.51–27.63)	21.03 (19.15–23.09)
Needle issue	2.42 (1.83–3.20)	4.38 (3.84–4.99)	2.76 (2.18–3.49)
Prescribed underdose	0.69 (0.39–1.22)*	3.28 (2.73–3.93)	6.59 (5.34–8.13)
Product communication issue	0.42 (0.06–3.00)*	15.62 (13.42–18.19)	16.58 (14.82–18.54)
Product storage error	1.88 (1.57–2.24)*	6.93 (6.55–7.34)	7.04 (6.61–7.50)
Underdose	14.17 (12.92–15.55)	20.93 (19.76–22.17)	21.12 (19.54–22.83)
Wrong technique in device usage process	1.11 (0.84–1.46)*	2.28 (2.00–2.59)	6.28 (5.53–7.13)
Wrong technique in product usage process	8.21 (7.73–8.72)	11.10 (10.72–11.49)	12.98 (12.40–13.59)
**Clinical adverse events**			
Accidental exposure to product	29.52 (27.82–31.32)	35.90 (34.70–37.14)	20.83 (19.91–21.79)
Alopecia	3.89 (3.57–4.23)	2.76 (2.55–2.99)	1.92 (1.67–2.20)*
Constipation	9.86 (9.18–10.60)	3.38 (3.15–3.62)	1.61 (1.43–1.824*
Headache	1.88 (1.74–2.03)*	2.68 (2.55–2.81)	1.78 (1.64–1.92)*
Injection site erythema	9.35 (8.42–10.38)	8.52 (7.93–9.17)	3.87 (3.46–4.34)
Injection site pain	8.74 (8.19–9.34)	15.23 (14.73–15.75)	11.20 (10.73–11.69)
Injection site pruritus	10.29 (9.05–11.70)	9.80 (8.99–10.69)	4.64 (4.04–5.34)
Injection site rash	11.99 (10.07–14.28)	7.96 (7.00–9.05)	4.01 (3.30–4.86)
Injection site reaction	10.21 (9.10–11.45)	7.11 (6.45–7.83)	3.96 (3.39–4.64)
Injection site swelling	9.52 (8.41–10.78)	8.63 (7.96–9.36)	3.90 (3.43–4.43)
Injection site urticaria	12.94 (10.76–15.56)	12.55 (11.18–14.08)	5.98 (5.03–7.11)
Medication overuse headache	56.92 (31.05–104.35)	13.33 (8.29–21.45)	2.91 (0.72–11.79)
Migraine	21.18 (19.69–22.79)	23.82 (22.74–24.95)	20.19 (18.95–21.51)
Therapeutic response shortened	18.65 (16.07–21.64)	10.95 (10.08–11.90)	3.77 (3.29–4.33)
Urticaria	2.03 (1.76–2.33)	1.44 (1.27–1.63)*	0.75 (0.61–0.93)*

*Events marked with an asterisk did not meet all criteria to be classified as a signal of disproportionate reporting (SDR). For an event to be classified as an SDR, it must meet three criteria simultaneously: (1) a number of reports ≥ 3; (2) a lower bound of the 95% CI of ROR greater than 1.0, and (3) a chi-square (*χ*^2^) value ≥ 4 (corresponding to *p* < 0.05). *NR* not reported

In sharp contrast, a sustained and significant decrease in reporting was observed for many well-established clinical AEs across the three periods (Table 1). The most dramatic decline occurred with “medication overuse headache,” where the ROR dropped sharply from 56.92 pre-pandemic to 13.33 during the pandemic, and further to 2.91 post-pandemic. A similar, consistent downward trend was seen for other known side effects, including “constipation” (ROR decreasing from 9.86 to 1.61) and “alopecia” (ROR decreasing from 3.89 to 1.92). This pattern of progressive decline was also evident for multiple injection site reactions, such as erythema, rash, and swelling.

**Fig. 1:**
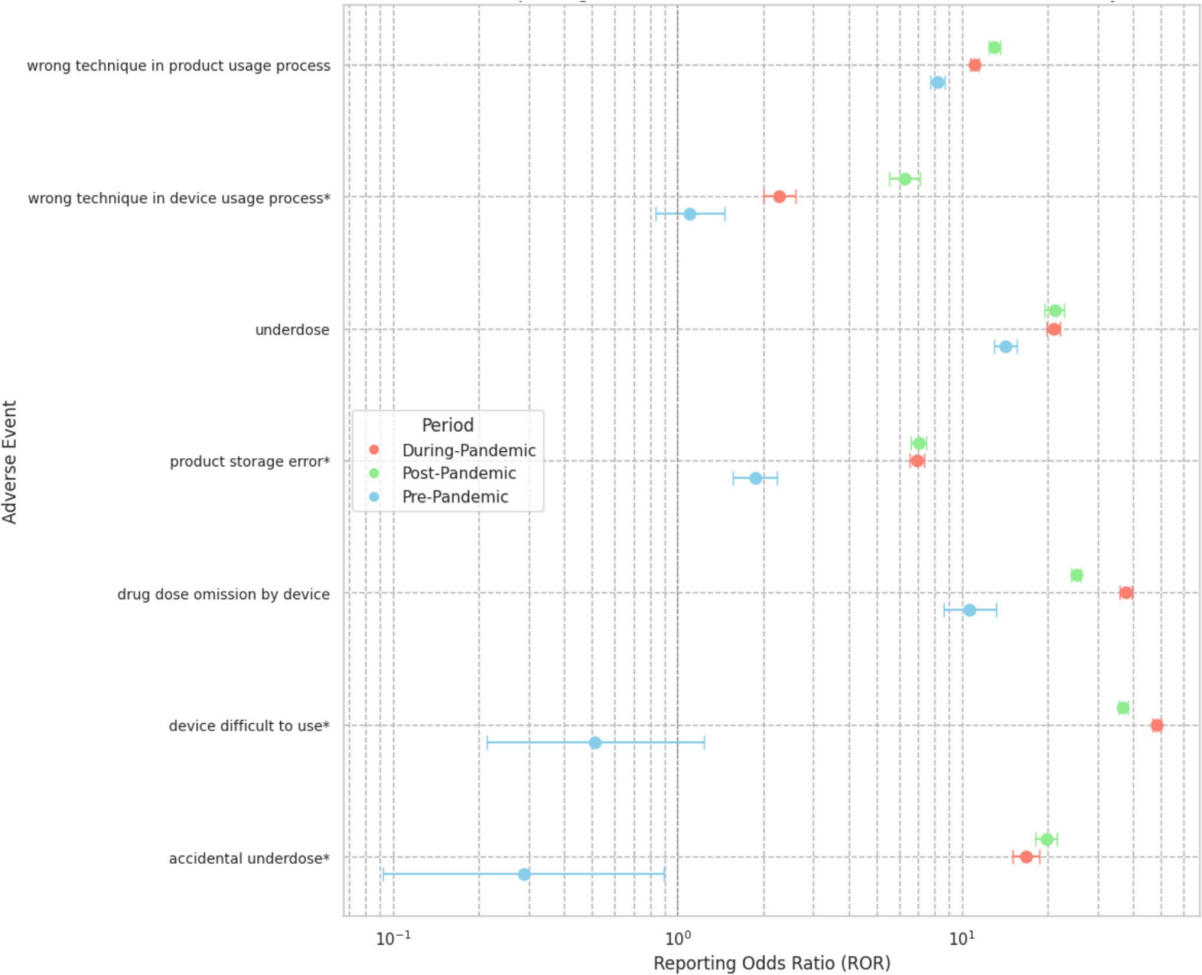
Increase in device-related and use-error adverse effects (AEs). The figure provides the longitudinal data for selected AEs that highlight the significant increase in reporting of issues related to device handling and administration errors, particularly during the pandemic. An asterisk (*) indicates that the Reporting Odds Ratio (ROR) for the pre-pandemic period did not meet all criteria to be classified as a signal of disproportionate reporting (SDR). For an event to be classified as an SDR, it must meet three criteria simultaneously: (1) a number of reports ≥ 3; (2) a lower bound of the 95% CI of ROR greater than 1.0, and (3) a chi-square (*χ*.^2^) value ≥ 4 (corresponding to *p* < 0.05)

**Fig. 2:**
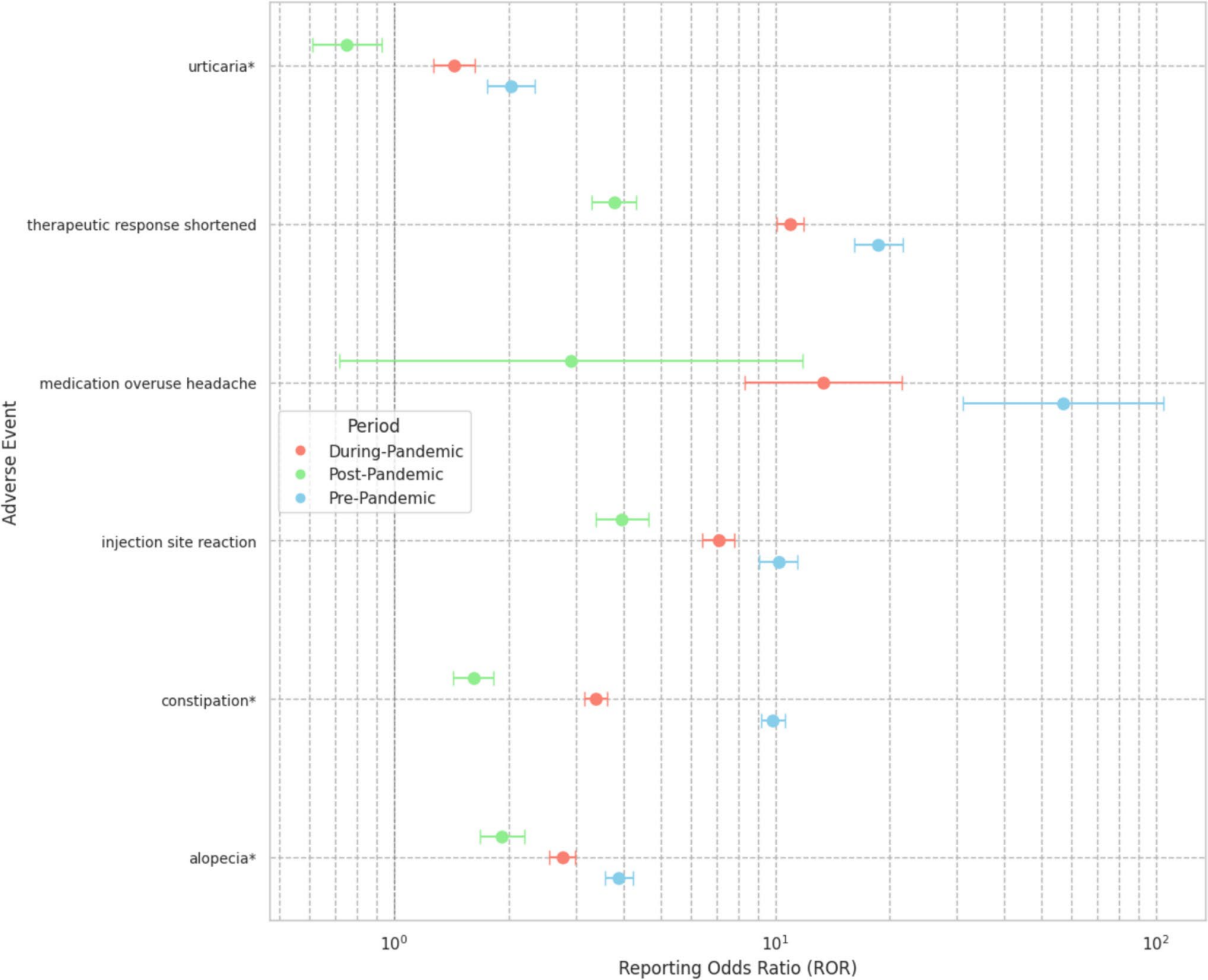
Decrease in reporting of known clinical AEs. The figure provides the longitudinal data for key clinical adverse events that show a consistent and significant decrease in reporting frequency across the three periods. An asterisk (*) indicates that the reporting odds ratio (ROR) for a specific period did not meet all criteria to be classified as a signal of disproportionate reporting (SDR). For “alopecia” and “constipation,” the asterisk refers to the post-pandemic period. For “urticaria,” the asterisk refers to both the during-pandemic and post-pandemic periods. For an event to be classified as an SDR, it must meet three criteria simultaneously: (1) a number of reports ≥ 3; (2) a lower bound of the 95% CI of ROR greater than 1.0, and (3) a chi-square (*χ*.^2^) value ≥ 4 (corresponding to *p* < 0.05)

To provide a longitudinal perspective, the temporal evolution of RORs for key AEs is visually summarized in forest plots. Given the large number of statistically significant signals detailed in Table 1, a representative subset of events was selected for graphical presentation to optimize visual clarity and avoid redundancy among clinically related terms (for example, displaying a broad category for injection site reactions rather than plotting each individual subtype). Figure 1 highlights trends for device-related AEs, illustrating both the initial surge and the sustained strengthening of specific signals, such as wrong technique in product usage process. Figure 2 shows the consistent decrease in reporting for well-established clinical AEs. Supplementary Fig. 2 in the Online Resource visualizes the emergence of new reporting signals, highlighting AEs that were not reported in the pre-pandemic baseline but became statistically significant signals in subsequent periods.

## Discussion

This pharmacovigilance analysis of AE reports for the CGRP monoclonal antibodies erenumab, fremanezumab, and galcanezumab suggests a potential significant and dynamic shift in reporting patterns across pre-pandemic, pandemic, and post-pandemic periods. Two prominent trends that emerged from the data were a statistically significant increase in device-related and administration-error AEs, and a concurrent decrease in the reporting of key clinical AEs. Reporting of key clinical AEs also showed significant temporal variations.

The surge in use-error reports appears to be temporally associated with the global disruption of healthcare services. The widespread shift to telemedicine and reduced in-person consultations created challenges in providing the hands-on patient training required for self-administered biologics (Shaver 2022), potentially contributing to fragmented instruction and a higher incidence of use errors. Furthermore, the demographic trend toward an older reporting population observed across the cohorts, as detailed in Supplementary Table 2 of the Online Resources, may have been a contributing factor. A lack of direct hands-on guidance could disproportionately affect older patients, who may be more susceptible to handling errors with novel self-injection devices. This observation is consistent with research reporting a higher proportion of medication errors linked to self-medication during the pandemic (Gras et al. 2021). While many of these SDRs declined post-pandemic with the restoration of routine clinical practice, the persistence and even strengthening of the “wrong technique in product usage process” SDR is a noteworthy observation. This could indicate a potential gap in patient education and raises the hypothesis that procedural skills, once poorly acquired, may not self-correct without proactive intervention. The increased reporting of “headache” is likely multifactorial. In addition to a potential pharmacodynamic effect, it may be influenced by pandemic-related stressors such as anxiety and altered routines, which are established migraine triggers. The negative psychological impact of the pandemic on individuals with chronic conditions is well documented (Burrows and Ellis 2022). In this context, worsening of the underlying disease may have been misattributed to the medication, a phenomenon potentially amplified by a nocebo effect driven by heightened health anxiety, a major concern during that period (Rief 2021).

Conversely, the analysis identified a consistent and significant decrease in reports of well-established clinical AEs such as “constipation.” This trend likely reflects a convergence of the natural maturation of the drugs’ safety profiles with pandemic-specific factors. While the Weber effect—characterized by a peak in reporting for recently approved products followed by a decline—predicts a natural reduction for these agents approved in 2018 (Hoffman et al. 2014), the magnitude of the decrease suggests that this trend was exacerbated by a shift in reporting priorities or “reporting fatigue.” Likely, strained healthcare providers (HCPs) and patients prioritized acute, device-related issues (which constitute an immediate barrier to treatment administration), over chronic well-known side effects. This aligns with findings of decreased reporting for unrelated drugs during the pandemic and analyses showing that the decline in spontaneous reporting was primarily driven by HCPs for non-serious events (Dörks al. 2021; Hauben and Hung 2021). This behavioral shift may have been compounded by the statistical “masking effect,” where the massive influx of COVID-19 vaccine reports inflated the database denominator, potentially suppressing the ROR for other drugs (Montes-Grajales et al. 2023). However, the empirical impact of this phenomenon is considered modest (Wang et al. 2010). Lastly, while the sustained decrease could reflect the natural maturation of the drugs’ safety profiles (the Weber effect), this classic pattern is not universally observed for recently approved drugs, suggesting a more complex interplay of factors (Hoffman et al. 2014). Finally, this selective decline in non-serious reporting helps reconcile the apparent paradox of the increased proportion of “life-threatening” outcomes observed in the post-pandemic cohort (4.2%). This rise likely does not reflect a true increase in clinical severity or toxicity, but rather a denominator effect. As the volume of reports describing common, non-serious events (such as constipation or injection site reactions) decreased due to the Weber effect and reporting fatigue, the relative proportion of serious outcomes—which are less prone to underreporting because of their gravity and regulatory requirements—mathematically increased within the dataset.

Our findings have several important clinical implications, positioning the pandemic as a critical stress test for managing self-administered therapies. First, they support the importance of exploring robust remote patient education and regular refresher training to reduce use-related errors and “skill decay” (Vlasblom et al. 2020). The persistence of use-error SDRs suggests that standard training practices may require re-evaluation. Second, clinicians should recognize the possible effects of major socio-environmental stressors on migraine and proactively counsel patients to manage expectations and avoid premature therapy discontinuation. Finally, these results suggest the importance of contextualizing pharmacovigilance data. It is plausible that signal detection algorithms may need to consider public health events, shifting from static analyses to dynamic models. This could involve integrating time-varying covariates related to healthcare access or utilizing change-point analysis to automatically detect structural breaks in reporting trends. This indicates a need for more adaptive and context-aware safety monitoring systems that can distinguish between genuine drug-related safety signals and artifacts caused by systemic disruptions (Tsuchiya et al. 2025).

The primary strength of this study lies in the use of the FAERS database, which provides a massive real-world dataset capable of capturing rare or unexpected adverse events that pre-authorization clinical trials often miss (Xie et al. 2025). Investigating this extensive repository within the unique context of the pandemic allows for the detection of dynamic shifts in SDRs that would otherwise remain unobserved. However, our findings must be interpreted in light of the inherent limitations of spontaneous reporting systems (SRS) (Wu et al. 2025). FAERS is designed for signal detection and cannot establish definitive clinical causality or calculate incidence rates due to the lack of denominator data regarding total patient exposure (Potter et al. 2025). Consequently, disproportionality measures reflect statistical associations within the database rather than absolute risk in the population (Cutroneo et al. 2024). Furthermore, the analysis is subject to established biases. First, underreporting is a systematic issue (Hazell and Shakir 2006) that was likely exacerbated by the overwhelming pressure on healthcare systems during the pandemic, specifically affecting the reporting of non-serious adverse events by overworked HCPs (Hauben and Hung 2021; van Hunsel and Kant 2025). Second, reporting patterns may be influenced by “notoriety bias” driven by external media or regulatory attention (de Boissieu et al. 2014). Third, the frequent incompleteness of granular clinical data—such as duration of therapy, history of device training, or patient comorbidities—limits a more detailed root-cause analysis of the observed trends, particularly regarding use-errors (Xie et al. 2025; Wu et al. 2025). Finally, we acknowledge the limitation regarding the aggregation of data from three different monoclonal antibodies. We strategically prioritized a class-level analysis to maximize statistical robustness—particularly for the pre-pandemic baseline—and to capture the systemic impact of pandemic-related healthcare disruptions on the shared route of self-administration. While this approach reflects the class-wide nature of the device-related challenges, it may mask drug-specific nuances or device-related differences that a granular, drug-by-drug analysis might reveal at the cost of reduced statistical precision. In addition, we must consider the impact of the massive influx of COVID-19 vaccine reports during the study period. This phenomenon, often called the “masking effect,” inflated the database denominator, potentially suppressing the ROR values for other drugs. Although the empirical impact of this effect is considered modest in some contexts (Wang et al. 2010), recent evidence suggests that the high volume of COVID-19 vaccine reports can significantly attenuate signals for non-vaccine drugs associated with the same adverse events (Micallef et al. 2023). This represents a structural limitation of disproportionality analysis during global health emergencies. Consequently, our results should be considered hypothesis-generating and require validation through rigorous clinical review and robust epidemiological studies.

## Conclusion

Our pharmacovigilance analysis of the FAERS database suggests that the COVID-19 pandemic was associated with significant shifts in the adverse event reporting profile for subcutaneously self-administered CGRP monoclonal antibodies. The marked increase in device-related reports highlights a potential vulnerability in the delivery of care for self-administered therapies during a public health crisis. Although some SDRs have diminished, the continued presence of specific use errors in the post-pandemic period may indicate a persistent gap in patient proficiency and device interaction. These findings support the urgent need for robust, remote-capable patient support and retraining systems, and highlight the importance of interpreting safety data within its socio-medical context. As national pharmacovigilance centers have noted, developing a resilient system that can rapidly scale up, supported by enhanced automation and transparent communication, is a key lesson from the COVID-19 pandemic (van Hunsel and Kant 2025). However, given the inherent limitations of spontaneous reporting systems, these conclusions should be interpreted as hypothesis-generating and warrant validation through controlled clinical studies.

## Supplementary Information

Below is the link to the electronic supplementary material.

Supplementary file 1 (DOCX 346 KB)

## Data Availability

All data generated or analysed during this study are included in this published article and its Online Resource.
